# Metabolomic Insights into MYMV Resistance: Biochemical Complexity in Mung Bean Cultivars

**DOI:** 10.3390/pathogens15010046

**Published:** 2025-12-31

**Authors:** Sudha Manickam, Veera Ranjani Rajagopalan, Madhumitha Balasubramaniam, Karthikeyan Adhimoolam, Senthil Natesan, Raveendran Muthurajan

**Affiliations:** 1 Department of Plant Biotechnology, Centre for Plant Molecular Biology and Biotechnology, Tamil Nadu Agricultural University, Coimbatore 641003, Tamil Nadu, India; rajaranji@gmail.com (V.R.R.); senthil_natesan@tnau.ac.in (S.N.); 2 Department of Plant Pathology, Agricultural College and Research Institute, Tamil Nadu Agricultural University, Madurai 625104, Tamil Nadu, India; madhu2sucess@gmail.com; 3 School of Agricultural Sciences, Karunya Institute of Technology and Sciences, Coimbatore 641114, Tamil Nadu, India; 4 Subtropical Horticulture Research Institute, Jeju National University, Jeju 63243, Republic of Korea; karthik2373@gmail.com

**Keywords:** green gram, resistant, susceptible, yellow mosaic, metabolite

## Abstract

Yellow Mosaic Disease (YMD) caused by mungbean yellow mosaic virus (MYMV, begomovirus) is one of the main causes of low mungbean (*Vigna radiata* L.) productivity, primarily in South Asia. Agroinoculation screening for MYMV resistance in mungbean cultivar VGGRU 1, an interspecific derivative of mungbean × rice bean and VRM (Gg)1 across replications, revealed VGGRU1 as highly resistant to MYMV infection. Gas chromatography mass spectrometry analysis was performed on the methanolic leaf extracts of susceptible and resistant genotypes, along with necessary controls. The metabolite profiling of the susceptible and resistant genotypes, along with controls, identified 121 discriminant metabolites belonging to 24 different classes of metabolites. A maximum number of 27 metabolites were accumulated in agroinoculated VGGRU1 alone. Metabolite profiles of VGGRU1 and VRM1 were clustered hierarchically and revealed substantial variations between the genotypes. Fold change revealed the upregulation of amino acids and phenol in the resistant genotype. The resistant genotype, VGGRU1, showed significantly higher levels of key defense-related metabolites, such as amino acids and phenolics. In this study, 18 significant VIP metabolites were identified, differentiating the resistant VGGRU1 and susceptible VRM (Gg)1 genotypes.

## 1. Introduction

Mungbean (*Vigna radiata* (L.) Wilczek), indigenous to India or the Indo-Burma region, ranks as the third most notable self-pollinated, short-duration grain legume crop, following chickpea and pigeon pea. The crop plays a vital role in mitigating protein malnutrition, particularly in developing countries, serving as an affordable and substantial source of dietary protein across Asia, most notably for impoverished populations. With a high percentage of easily digestible, top-quality protein (24%) and minimal flatulence, along with its elevated iron content (40–70 ppm), mungbean is an excellent addition to balanced diets. Additionally, mungbean sprouts are highly valued in Asian cuisine not only for their seeds but also for their rich content of vitamin C and folate [1,2,3].

Mungbean is susceptible to pests and diseases, including Yellow Mosaic Disease (YMD) caused by mungbean yellow mosaic virus (MYMV), which results in yield losses of up to 100% in the Indian subcontinent and South-East Asia. Three distinct begomoviruses have been identified as the primary pathogens responsible for YMD: MYMV, mungbean yellow mosaic India virus (MYMIV), and horse gram yellow mosaic virus (HgYMV). In India, YMD is attributed to MYMV/MYMIV, and according to reports, MYMV is predominantly found in southern India, whereas MYMIV is prevalent in the northern, central, and eastern regions of the country [4,5]. Apart from mungbean, YMD also impacts other leguminous crops including cowpea, horse gram, pigeon pea, French bean, black gram, moth bean, Lima bean, and soybean [6,7]. The virus spreads via the whitefly’s proboscis in a persistent and circulative manner, infiltrating the host’s phloem cells [8]. Whiteflies can inflict significant damage to a plant with a single attack, posing challenges for effective management. The two indigenous cryptic species, Asia II-1 and Asia II-8, are reported to be prevalent in different regions of India [9]. Mungbean plants infected within three weeks of sowing may have a reduced yield up to 85%. The initial symptoms after infection appear as small yellow lesions on the young leaves with less intensity [10], evolving into a mosaic pattern of yellowing, eventually leading to complete chlorosis, desiccation, and senescence of the foliage [11]. The affected mungbean plants exhibit reduced pod size and chlorotic leaves, leading to diminished photosynthetic efficiency and a consequential decrease in overall yield.

The use of pesticides to manage YMD (for controlling whiteflies) was initially considered effective, but the disease is spreading due to the development of pesticide-resistant vectors. Additionally, excessive chemical usage has led to detrimental effects on the environment and human health [12]. However, utilizing mungbean cultivars resistant to MYMV has long been recognized as an effective and cost-efficient strategy for virus control. Unfortunately, confirming resistance through field screening presents challenges due to the non-consistence of MYMV symptoms in field conditions due to factors like environmental variations, whitefly genotypes, and host characteristics. This variability can impede infection development in the field, thus making it difficult to identify truly resistant lines. Consequently, significant research efforts have focused on screening mungbean germplasms using agroinoculation, an innovative strategy that utilizes the tumor-inducing plasmid of *Agrobacterium tumefaciens* to introduce infectious viral clones into plants. This approach induces MYMV symptoms through encapsidation and replication, and its effectiveness in screening has been successfully demonstrated by some researchers [13,14]. Still, a limited number of mungbean lines demonstrated resistance to YMD, although the majority exhibited low yields [15,16,17]. Reports by Khattak [18] and Akhtar [19] indicate that mungbean germplasm possessing high yield potential tends to be susceptible to YMD. However, achieving high-yielding mungbean varieties that are resistant to YMD necessitates a comprehensive understanding of genetic variations in metabolite accumulation. Non-targeted metabolomics enables the profiling of a wide range of secondary metabolites in plants. In this study, a non-targeted metabolomics approach was used to analyze the metabolomic profiles of high-yielding susceptible cultivar VRM (Gg)1 and resistant cultivar VGGRU1. Investigating the intricate metabolome of VGGRU1 and VRM (Gg)1 would enable us to understand and map these traits for utilization in breeding programs.

## 2. Materials and Methods

### 2.1. Plant Material

MYMV-resistant mungbean cultivar VGGRU 1 (interspecific derivative of mungbean × rice bean) and susceptible cultivar VRM (Gg)1 were used in this study. Seed materials were obtained from Agricultural Research Station, Tamil Nadu Agricultural University, located in Virinjipuram, Tamil Nadu.

### 2.2. Agroinoculation

**Figure 1:**
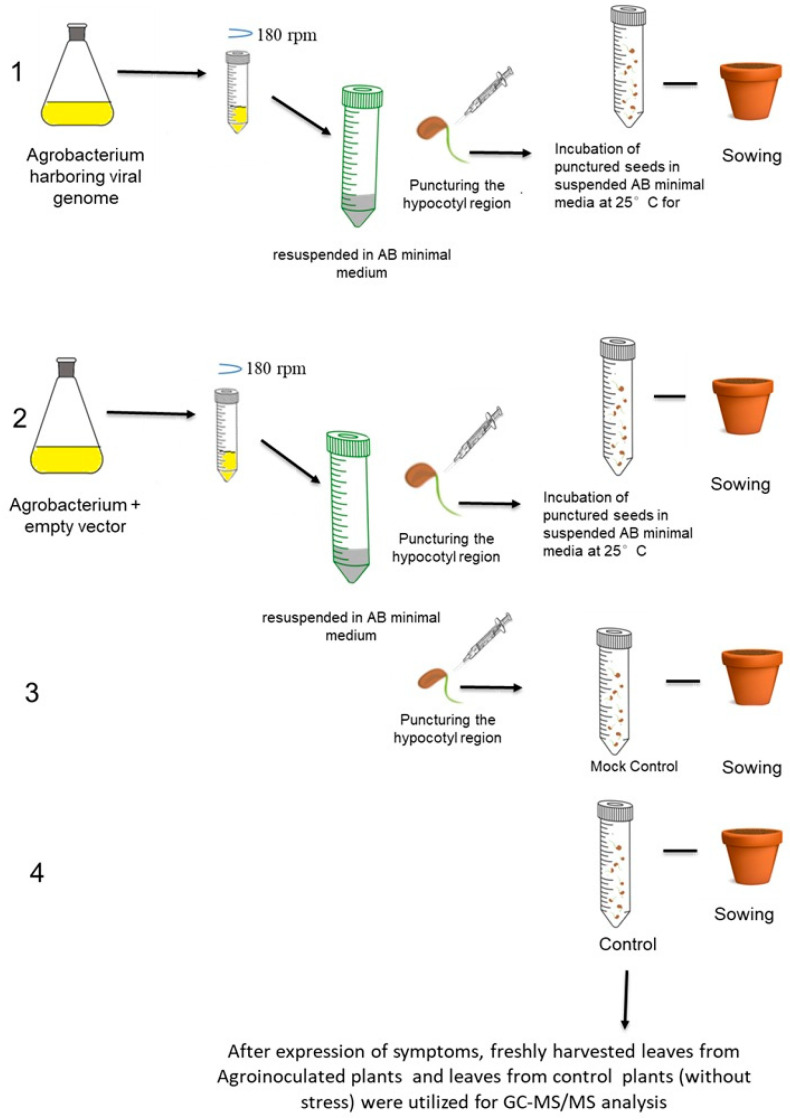
Schematic representation of the experimental design: 1. inoculation of seeds with *Agrobacterium* + MYMV genome cloned vector, 2. inoculation of seeds with *Agrobacterium* + empty vector, 3. mock control, and 4. untreated control followed by sowing. Experiment was performed for both VGGRU1 and VRM (Gg)1, and symptomatic, healthy leaves were subjected for GC-MS analysis.

The study utilized the infectious clone that was developed from our previous study [20], derived from the MYMV genome. Agroinoculation was performed on 2-day-old sprouted seeds of both resistant and susceptible lines, along with the empty vector, mock control, and untreated control following a previously established protocol by [21]. The agroinoculated plants were cultivated in a growth chamber set at 25 °C, with 60–70% relative humidity under a 16/8 h photoperiod at the department of biotechnology, center for plant molecular biology and biotechnology, Coimbatore. Hoagland’s solution (KNO_3_-6 mM, Ca(NO_3_)_2_·4H_2_O-4 mM, KH_2_PO_4_-1 mM, MgSO_4_·7H_2_O-2 mM, H_3_BO_3_-46 µM, MnCl_2_·4H_2_O-9 µM, ZnSO_4_·7H_2_O-0.8 µM, CuSO_4_·5H_2_O-0.3 µM, (NH_4_)_6_Mo_7_O_24_·4H_2_O-0.125 µM, and Fe-EDTA-100 µM) was applied twice a week to promote plant growth, and symptom development was monitored starting from the 15th day post-inoculation on trifoliate leaves. The presence of yellow mosaic symptoms at a specific time was recorded as susceptible, while their absence indicated resistance to the disease. Uninoculated plants of each genotype were kept as controls for comparison. Following the expression of symptoms, freshly harvested leaves were utilized for GC-MS/MS analysis. Figure 1 represents the schematic figure illustrating the experimental design.

### 2.3. Extraction and Mass Spectrometric Analysis of Secondary Metabolites

The VRM (Gg) 1 and VGGRU-1 treated with *Agrobacterium* + MYMV genome cloned vector along with the untreated control were used for further study. Secondary metabolites were extracted from the fresh leaves of VGGRU1 control, VGGRU1 infected with MYMV genome, VRM (Gg)1 control, and VRM (Gg1) infected with MYMV genome using the previously described Soxhlet extraction method [22]. Three biological replications were used in each sample. The leaves were first pulverized using liquid nitrogen in a mortar and pestle. Approximately 25 mg of the powdered leaf sample was then soaked in 100% methanol (HPLC grade) overnight. The mixture was heated in a water bath at 70 °C for 10 min, followed by centrifugation at 13,000× *g* for 10 min at 4 °C. The supernatant was collected and filtered through a 0.2-micron filter. The filtered extracts were concentrated using a vacuum evaporator, and 1 mL of the concentrated filtrate was transferred into vials for analysis by mass spectrometry. For mass spectrometric analysis, a GC-MS/MS instrument (Perkin Elmer Inc., Akron, OH, USA) equipped with a DB-5 MS capillary standard non-polar column (30 m length, inner diameter: 0.25 mm, film thickness: 0.25 μm, Perkin Elmer Inc., Akron, OH, USA), located in the Department of Agricultural Microbiology at Tamil Nadu Agricultural University, Coimbatore, India, was utilized. One microliter of the methanolic extract sample was injected into the GC-MS/MS system with helium as the carrier gas. The peaks were detected over a 30 min run time. Towards the end of each run, a high temperature (260 °C) was maintained for approximately 5 min, followed by syringe washing with methanol (three times) and equilibration (2–3 min) to prevent contamination. The GC-MS/MS analysis involved scanning a mass range of 50–1000 *m*/*z* with a fragmentation energy of 70 eV. Precursor ions were isolated using an isolation window of 10 *m*/*z*. The raw mass spectra obtained were converted to ABF format using an ABF converter (https://www.reifycs.com/abfconverter/, accessed on 25 November 2021) for further analysis.

### 2.4. Analysis and Pathway Mapping Using Statistical Methods

The processing and annotation of spectral peaks were conducted using MS-DIAL [22]; principal component analysis (PCA) and partial least squares discriminant analysis (PLS-DA) were performed using MetaboAnalyst 5.0 (https://www.metaboanalyst.ca/, accessed on 29 November 2021) [23], where missing values were replaced by 1/5 of the minimum positive values. To identify significant metabolites distinguishing VGGRU1 and VRM (Gg)1, these metabolites were mapped onto metabolic pathways using Metabo Analyst 5.0 [23]. Significant pathways showing differences between VGGRU1 and VRM (Gg)1 were identified using the Kyoto Encyclopedia of Genes and Genomes (KEGG) database [24].

## 3. Results

VGGRU1, a high-level MYMV-resistant derivative of *Vigna radiata* × *Vigna umbellata*, whereas VRM (Gg)1 is a MYMV-susceptible genotype (Table 1).

### 3.1. Agroinoculation Screening and Symptom Development

**Figure 2:**
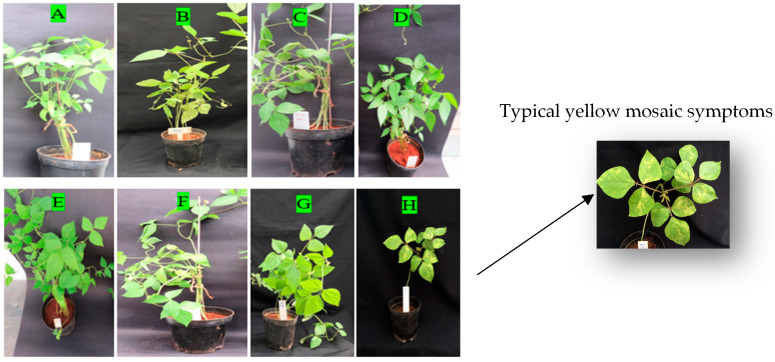
(**A**) VGGRU-1 treated (*Agrobacterium* + empty vector), (**B**) VGGRU-1 (mock control), (**C**) VGGRU-1 untreated control, (**D**) VGGRU-1 treated (*Agrobacterium* + MYMV genome cloned vector), (**E**) VRM (Gg) 1 treated (Agrobacterium + empty vector), (**F**) VRM (Gg) 1 (mock control), (**G**) VRM (Gg) 1 untreated control, and (**H**) VRM (Gg) 1 treated (*Agrobacterium* + MYMV genome cloned vector).

Following MYMV inoculation on two mungbean genotypes, VRM (Gg) 1 and VGGRU-1, in susceptible mungbean genotype VRM (Gg) 1, a typical mosaic symptom was noted. Conversely, the resistant genotype VGGRU-1 did not exhibit any signs until 40 DPI (Figure 2). The plants inoculated with (*Agrobacterium* + empty vector), mock control with buffer alone, and the untreated control for both the susceptible and resistant genotype did not show any typical yellow mosaic symptoms across replications.

### 3.2. Metabolite Profiles of the Susceptible and Resistant Mung Bean Genotypes

**Figure 3:**
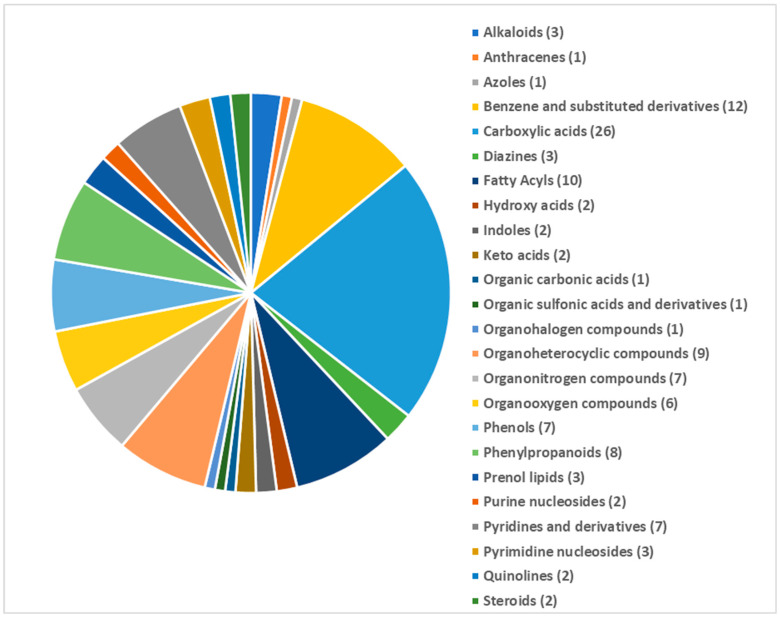
Classification of 121 metabolites into different metabolite classes expressed in the metabolite profiling of the control VGGRU1, treated VGGRU1, control VRM (Gg)1, and treated VRM (Gg)1.

The metabolite profiling of the control VGGRU1, treated VGGRU1, control VRM (Gg)1, and treated VRM (Gg)1 identified 121 discriminant metabolites belonging to 24 different metabolite classes (Figure 3).

**Figure 4:**
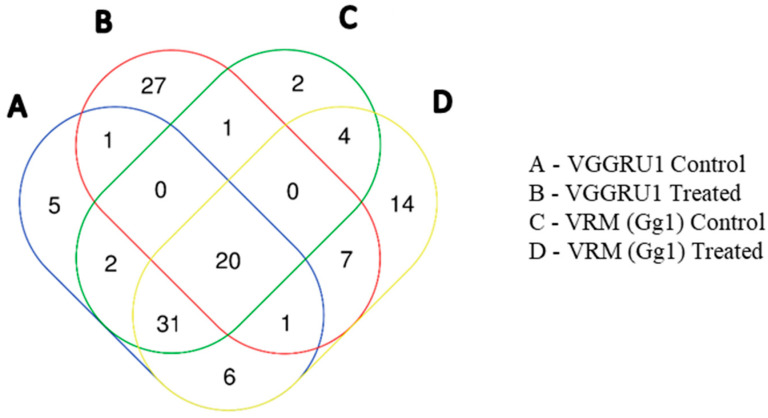
Venn diagram showing accumulation of number of metabolites in VGGRU1 and VRM (Gg)1.

A total of 20 metabolites were observed in all the four samples. A maximum number of 27 metabolites were accumulated in the VGGRU1 treated group alone (Figure 4).

**Figure 5:**
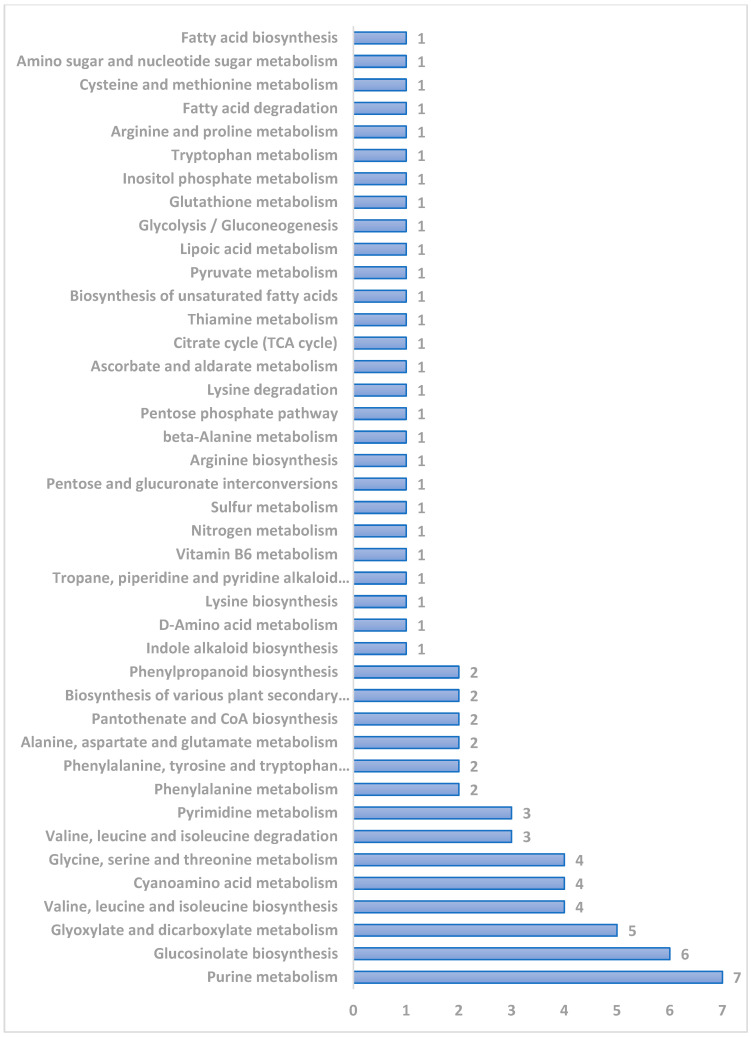
Mapping of 24 different metabolite classes against KEGG metabolic pathways. (Numbers at the end of the bar indicate total number of metabolites involved in each pathway).

Mapping of all 24 different metabolite classes against KEGG metabolic pathways revealed 41 different sub-pathways (Figure 5 and Supplementary Table S1).

Many of the metabolites are mapped onto purine metabolism (7), glucosinolate metabolism (6), glyoxylate and dicarboxylate metabolism (5), valine, leucine, and isoleucine biosynthesis (4), cyanoaminoacid metabolism (4), and glycine, serine, and threonine metabolism (4).

### 3.3. Chemometric Analysis

**Figure 6:**
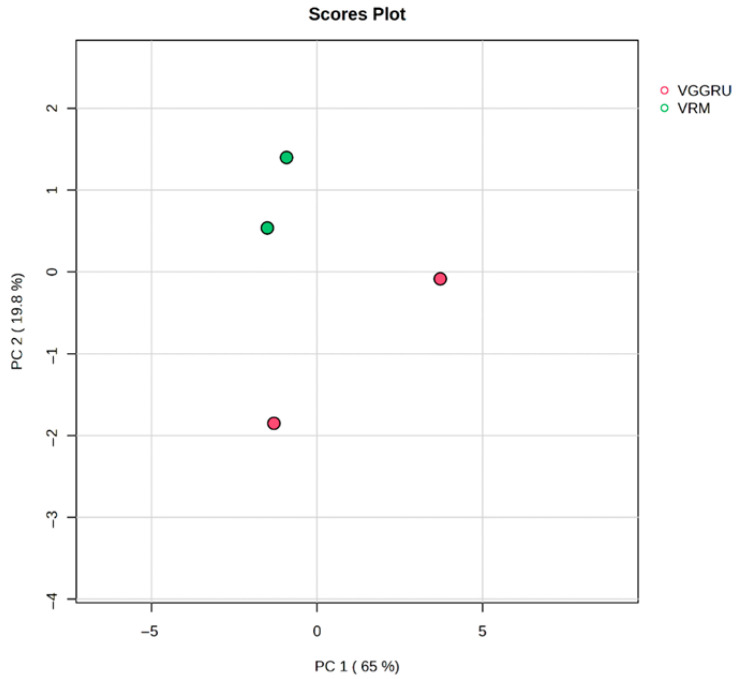
Score plots of principal component analysis of VGGRU1 and VRM (Gg1) metabolites.

Metabolite profiles of VGGRU1 and VRM1 were clustered hierarchically and revealed substantial variations between the genotypes. Multivariate analysis *viz* principal component analysis (PCA) and partial least square-discriminant analysis (PLS-DA) was performed to identify the metabolite variation between VGGRU1 and VRM (Gg)1. A principal component analysis (PCA) estimated the metabolic differences between the resistant VGGRU1 and the susceptible VRM (Gg)1. The PCA results revealed that the first component, PC1, accounted for 65.3% of the variance, while PC2 explained 19.6% of the total variance of 84.9% (Figure 6).

**Figure 7:**
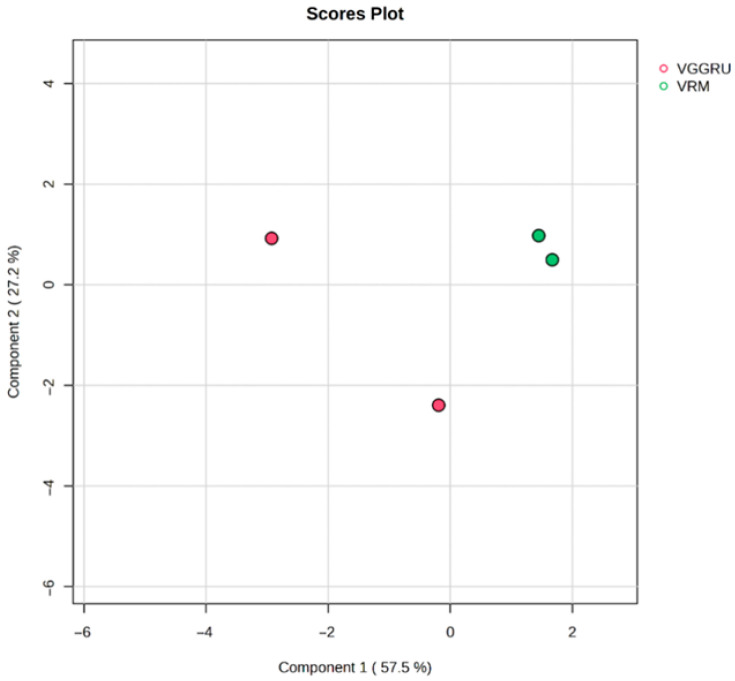
Partial least square-discriminant analysis (PLS-DA) score plot for metabolite profiles between VGGRU1 and VRM (Gg1).

The partial least square-discriminant analysis (PLS-DA) results revealed similar differences, with a cumulative variance of 89.3% (component 1 explains 61.3% and component 2 explains 28% of the variance) (Figure 7).

**Figure 8:**
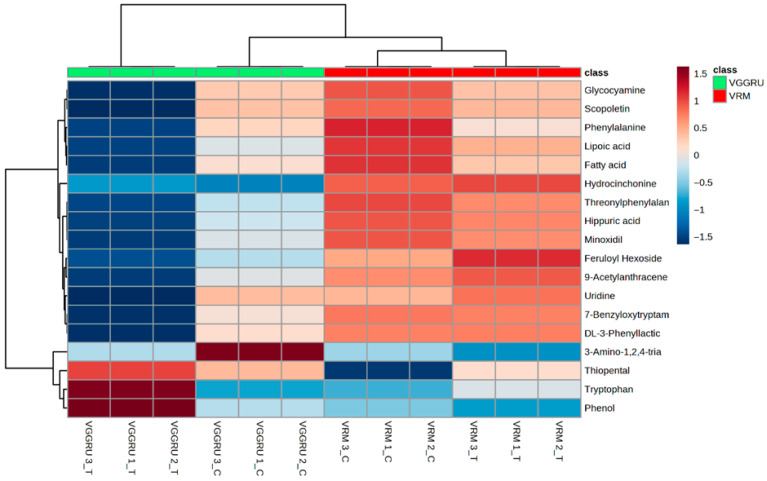
Heat map of PLS-DA VIP metabolites discriminating VGGRU1 and VRM (Gg1) (scale indicates chromatogram peak intensity from low (blue) to high (red)).

Out of 121 annotated metabolites, the PLS-DA identified 18 significant VIP metabolites differentiating the resistant VGGRU1 and susceptible VRM (Gg)1 genotypes with a VIP score of more than 1 (Figure 8).

### 3.4. Hierarchial Clustering and Fold Change Analysis

**Figure 9:**
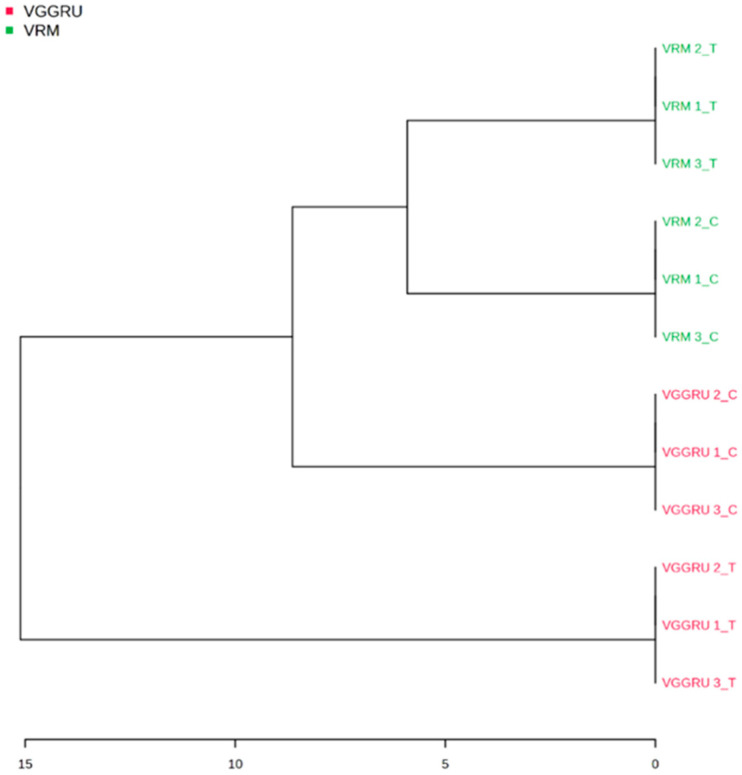
Clustering based on metabolite profiles.

In order to comprehend the metabolic diversity between the VGGRU1 control, VGGRU1 treated, VRM (Gg1) control, and VRM (Gg1) treated groups, hierarchical clustering was used. As a result, the data was grouped into two major clusters: the first cluster consisted of the VGGRU1 treated, VRM control, and VRM treated groups, while the second cluster was composed of the VGGRU1 treated group alone (Figure 9).

Eight upregulated metabolites and thirteen downregulated metabolites were found when the abundance ratios of the metabolites found in the VGGRU1 and the VRM (Gg1) were examined. While polyphenols, essential amino acids, phenolic acids, and alkaloids are among the downregulated metabolites, amino acids and phenol made up most of the upregulated metabolites (Table 2).

### 3.5. Pathway Analysis

**Figure 10:**
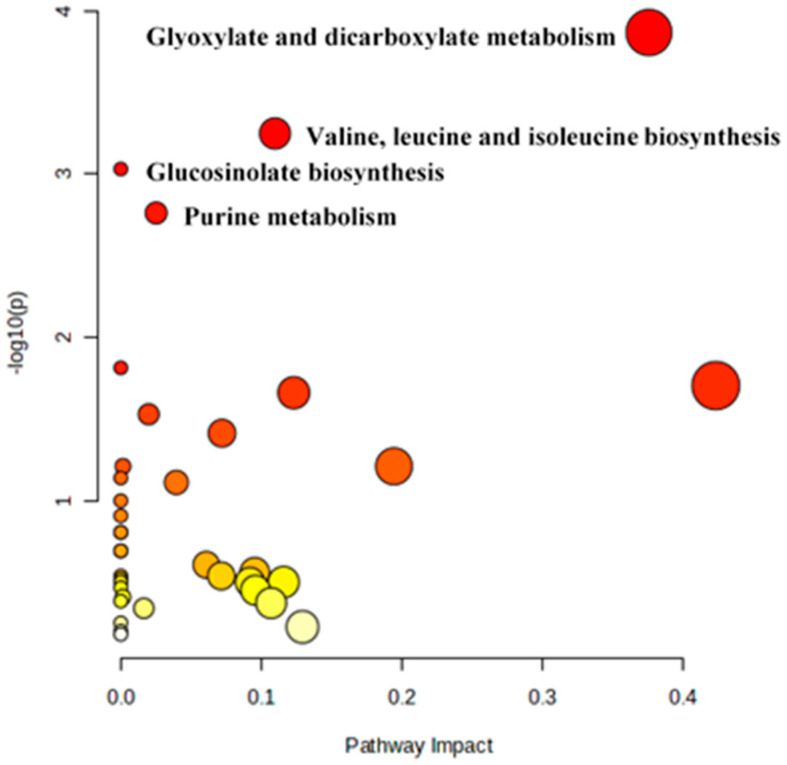
Major pathway discriminating VGGRU1 and VRM (Gg1).

Pathway mapping against the KEGG database identified four significant pathways, which showed an FDR of less than 0.05 (Table 3, Figure 10). Glyoxylate and dicarboxylate metabolism showed the highest –log10(*p*) value of 3.866, followed by valine, leucine, and isoleucine biosynthesis (3.248), glucosinolate biosynthesis (3.0302), and purine metabolism (2.7611).

## 4. Discussion

MYMV and MYMIV are the primary causes of YMD in mungbean and its relatives, such as cowpea and black gram [25]. Crop loss of MYMV might vary from 40 to 100 percent, depending on the cultivar and infection duration [26]. The begomoviruses that cause YMD have been thoroughly reported in several legume crops in India [27,28,29,30,31]. The breeding program for disease resistance in legume crops against YMD may be greatly impacted by the emergence of MYMV-resistant varieties.

Identification of MYMV resistance through visual observation is not often easy, because the symptoms are not always evident in practice. A 100% infection rate cannot be guaranteed by whitefly-based inoculation since MYMV is not mechanically transferred. In the past, our team has used rolling circle amplification (RCA) to define a distinct strain of MYMV and created an agroinfectious construct with a bipartite genome (DNA A + DNA B) [14,20]. This agroinoculation approach offers systemic infection in the plants [32]. The primary advantage of agroinoculation is that, in contrast to spontaneous infections, it results in uniform disease signs that are easier to quantify [33]. The search for MYMV resistance often fails in legume breeding; however, numerous studies have demonstrated a wild legume rice bean (*V. umbellata*) that possesses notable resistance to YMV infection [33,34]. Therefore, in the present study we have chosen an MYMV cultivar, which is an interspecific derivative of *Vigna radiata* (mungbean) × *Vigna umbellata* (rice bean) that has proven high-level resistance against MYMV in the field, as well agroinoculation and susceptible cultivar VRM (Gg)1 [20]. The infectivity was confirmed across replications.

Advanced molecular methods have been developed to analyze the transcriptomes, proteomes, and metabolomes of crop plants [35]. The present study aimed to unravel the metabolome complexity of mungbean resistant VGGRU1 and susceptible cultivar VRM (Gg)1. There have been a few attempts to measure the amounts of proteins, carbohydrates, and antioxidants in the leaves of VGGRU1 and VRM (Gg)1, but no comprehensive study has examined the makeup of the secondary metabolites in these leaves [36]. In this study, non-targeted metabolomic profiling was carried out in the MYMV-treated and control plants of VGGRU1 and VRM (Gg)1.

The GC-MS/MS metabolite profiling of the control VGGRU1, treated VGGRU1, control VRM (Gg)1, and treated VRM (Gg)1 revealed a total of 121 discriminant metabolites belonging to 24 different metabolite classes. Of these 121 metabolites, a total of 20 metabolites were observed in all four samples, and a maximum number of 27 metabolites were accumulated in the VGGRU1-treated group alone. Interestingly, mapping of all 24 different metabolite classes against KEGG metabolic pathways revealed 41 different sub-pathways. The PCA results revealed a unique separation between the resistant and susceptible cultivars based on the metabolites. The fold change analysis revealed that the defense metabolites such as the phenolic compound, amino acids and fatty acid ester were upregulated, whereas stress-related amino acids and sugars were downregulated in the leaves of the mungbean cultivar. The PLS-DA identified 18 significant VIP metabolites belonging to coumarin, amino acids, sugars, alkaloids, antioxidants, and carboxylic acids differentiating the resistant VGGRU1 and susceptible VRM (Gg)1 genotypes, which are known for their antimicrobial and defense responses. Fold change analyses also confirmed the abundance of polyphenols, essential amino acids, phenolic acids, and alkaloids.

The accumulation of a few defenses related to biomolecules such as Tryptophan, Phenol, 3-Amino-1,2,4-triazole, Cyprodinil, 2-Amino-3-methylimidazo (4,5-f) quinoline, Diethyldiallylmalonate, glycine, and Hypoxanthine was observed. Tyrosine (Tyr), phenylalanine (Phe), and Tryptophan (Trp or W), three polar amino acids with an indole ring, are members of the aromatic amino acid group. Tryptophan is a substance that can be found free or in proteins [37]. It acts as a precursor to a wide range of secondary metabolites such as phytoalexins, indole glucosinolates, the plant hormone auxin, serotonin, and melatonin [38,39,40]. Many plant families, including *Brassicaceae*, *Fabaceae*, *Solanaceae*, *Vitaceae*, and *Poaceae*, produce phytoalexins as a defense mechanism against pathogens [41,42]. Therefore, the accumulation of Tryptophan in VGGRU1 might act as a precursor in phytoalexin production, which in turn activates a wide range of molecules such as terpenoids, glycosteroids, and alkaloids, which possess antioxidant activity [43,44,45].

Glycine regulates the trans membranous trafficking of Ca++ and is crucial for preserving the intracellular concentration of one carbon group. Ionized calcium functions as a cytoprotectant and is essential for cell signal transmission [46]. Additionally, glycine contributes to the synthesis of glycine-rich proteins, the expression of which is controlled by stress stimuli from the outside world. Thus, resistance may have been conferred by the buildup of glycine, which may have aided in efficient signal transmission in VGGRU1.

Phenolic compound functions are diverse in plants, ranging from roles in growth and development to providing plant defense, i.e., antimicrobial activity [47]. During plant organism interactions, phenolics are significantly greater in plants fed upon by insects or colonized by microorganisms as compared to untouched plants [48]. In VGGRU1, the overexpression of phenols is noted, and this may have been a source of protection from virus-induced damages, thereby conferring resistance; in addition, phenolic production was often considered to be associated with SA-resistance and JA responses that induce plant defense [49,50,51,52,53].

A strong antioxidant, scopoletin is a member of the coumarin class of secondary metabolites [54]. Depending on the genotype, age, plant part, and physiological condition, the endogenous level of scopoletin frequently correlates with the extent of disease susceptibility or resistance in various plants [55,56,57,58,59,60,61]. Upregulation of scopoletin in VRM (Gg)1 may induce its susceptibility.

Under stress conditions, many plant species accumulate proline as an adaptive response to adverse conditions [62]. The proline content increases upon pathogen infection [63], possibly to decrease the rate of disease progression as a part of basal defense response; the upregulation of proline in VRM (Gg)1 combatted the pathogen and failed to resist the invasion and developed characteristic YMV symptoms.

Stress-responsive metabolites, like amino acids and alkaloids, were increased in VRM (Gg)1, but they were unable to fend off the pathogen invasion and instead displayed the telltale signs of YMV. A few inadequately filled pods and new flowers appearing up until harvest are signs that the plant’s stress-responsive function may not be functioning or that certain metabolites may have been redirected in the plant’s developmental process.

## 5. Conclusions

The study highlights VGGRU1, an interspecific mungbean derivative, as highly resistant to MYMV, with metabolomic profiling revealing distinct biochemical differences from the susceptible genotype VRM (Gg)1. Gas chromatography-mass spectrometry identified 121 metabolites, with VGGRU1 uniquely accumulating 27 compounds upon MYMV agroinoculation. Key defense-related metabolites, including amino acids and phenolics, were significantly upregulated in the resistant genotype. Multivariate analyses (PCA and PLS-DA) confirmed clear separation between the genotypes, identifying 18 significant VIP metabolites, while KEGG pathway mapping revealed four enriched pathways. These findings suggest a strong metabolic basis for MYMV resistance in VGGRU1 and provide useful targets for resistance breeding.

## Figures and Tables

**Table 1:** Details of the agronomic characteristics of mungbean genotypes VGGRU1 and VRM (Gg)1.

Cultivar	Origin	Days of Maturity	Pedigree	Features
VGGRU1	TNAU, Coimbatore, India	60–75 days	High-level MYMV-resistant derivative of *Vigna radiata* × *Vigna umbellata*	MYMV resistant
VRM (Gg)1	TNAU, Coimbatore, India	56–67 days	Pure line selection from K 851	MYMV susceptible

**Table 2:** Abundance ratio of 21 metabolites showing more than 2-fold change between VGGRU1 and VRM (Gg)1.

S. No	Compound Name	Fold Change	Log 2 (Fold Change)
1.	Tryptophan	17.102	4.0961
2.	3-Amino-1,2,4-triazole	15.478	3.9522
3.	Phenol	6.4135	2.6811
4.	Cyprodinil	3.4521	1.7875
5.	2-Amino-3-methylimidazo(4,5-f) quinoline	2.7709	1.4703
6.	Diethyldiallylmalonate	2.5143	1.3301
7.	Glycine	2.4229	1.2768
8.	Hypoxanthine	2.0405	1.0289
9.	Scopoletin	0.49484	−1.015
10.	Proline	0.4582	−1.1259
11.	Phenylalanine	0.38899	−1.3622
12.	DL-3-Phenyllactic acid	0.36861	−1.4398
13.	7-Benzyloxytryptamine	0.3423	−1.5467
14.	9-Octadecanoic acid	0.33494	−1.578
15.	9-Acetylanthracene	0.25516	−1.9705
16.	Minoxidil	0.24339	−2.0387
17.	Lipoic acid	0.24194	−2.0473
18.	Hippuric acid	0.20954	−2.2547
19.	Hydrocinchonine	0.18042	−2.4706
20.	Threonylphenylalanine	0.17216	−2.5382
21.	Feruloyl Hexoside	0.13926	−2.8442

**Table 3:** List of significant metabolic pathways discriminating VGGRU1 and VRM (Gg)1.

S. No	Pathway	Raw *p* Value	−log(*p*)	FDR
1.	Glyoxylate and dicarboxylate metabolism	0.000136	3.866	0.012388
2.	Valine, leucine, and isoleucine biosynthesis	0.000565	3.248	0.025707
3.	Glucosinolate biosynthesis	0.000933	3.0302	0.028296
4.	Purine metabolism	0.001734	2.7611	0.039438

## Data Availability

All the data are available in the main text itself.

## Supplementary Materials

The following supporting information can be downloaded at: https://www.mdpi.com/article/10.3390/pathogens15010046/s1, Table S1: Metabolites present in each identified pathway; Figure S1: Graphs denote qRT-PCR-based validation of DEGs of mungbean leaves infected with mungbean yellow mosaic virus. The y-axis denotes relative fold change compared to control and infected. Data represent means ± SD of three replicates.
