# Supplementary material for: Metabolomic Insights into MYMV Resistance: Biochemical Complexity in Mung Bean Cultivars
Source: Pathogens. 2025 Dec 31;15(1):46. doi: 10.3390/pathogens15010046 (PMC12845075; doi:10.3390/pathogens15010046)
Supplement: Supplementary file 1 [file pathogens-15-00046-s001.zip › pathogens-3927019-supplementary.pdf]

Supplementary Table S1. Metabolites present in each identified pathway

S. No	Pathway	Number of metabolites	Metabolite name
1.	Purine metabolism	7	L-Glutamine; Hypoxanthine; Urate, Inosine; Guanosine, Glyoxylate, Glycine
2.	Glucosinolate biosynthesis	6	L-Methionine; L-Phenylalanine, L-Tryptophan, L-Isoleucine, 3-Methyl-2-oxobutanoic acid; (S)-3-Methyl-2-oxopentanoic acid
3.	Glyoxylate and dicarboxylate metabolism	5	Glyoxylate; Glycine; L-Glutamine, Acetate; Citrate
4.	Valine, leucine and isoleucine biosynthesis	4	L-Threonine; (S)-3-Methyl-2-oxopentanoic acid; 3-Methyl-2-oxobutanoic acid; L-Isoleucine;
5.	Cyanoamino acid metabolism	4	L-Asparagine, Glycine, L-Phenylalanine; L-Glutamine
6.	Glycine, serine and threonine metabolism	4	Glycine, L-Threonine; Glyoxylate, L-Tryptophan
7.	Valine, leucine and isoleucine degradation	3	S)-3-Methyl-2-oxopentanoic acid; 3-Methyl-2-oxobutanoic acid; L-Isoleucine;
8.	Pyrimidine metabolism	3	L-Glutamine, Uridine; 5,6-Dihydrouracil;
9.	Phenylalanine metabolism	2	L-Phenylalanine; Phenylacetic acid
10.	Phenylalanine, tyrosine and tryptophan biosynthesis	2	L-Tryptophan; L-Phenylalanine
11.	Alanine, aspartate and glutamate metabolism	2	L-Asparagine, L-Alanine; L-Glutamine
12.	Pantothenate and CoA biosynthesis	2	5,6-Dihydrouracil, 3-Methyl-2-oxobutanoic acid
13.	Biosynthesis of various plant secondary metabolites	2	L-Methionine, Scopoletin
14.	Phenylpropanoid biosynthesis	2	L-Phenylalanine, 5-Hydroxyferulic acid;
15.	Indole alkaloid biosynthesis	1	L-Tryptophan;
16.	D-Amino acid metabolism	1	L-Lysine
17.	Lysine biosynthesis	1	L-Lysine
18.	Tropane, piperidine and pyridine alkaloid biosynthesis	1	L-Phenylalanine;
19.	Vitamin B6 metabolism	1	L-Glutamine
20.	Nitrogen metabolism	1	L-Glutamine;
21.	Sulfur metabolism	1	Acetate
22.	Pentose and glucuronate interconversions	1	D-Glucuronate;

S. No	Pathway	Number of metabolites	Metabolite name
23.	Arginine biosynthesis	1	L-Glutamine
24.	beta-Alanine metabolism	1	5,6-Dihydrouracil
25.	Pentose phosphate pathway	1	D-Ribose
26.	Lysine degradation	1	L-Lysine
27.	Ascorbate and aldarate metabolism	1	D-Glucuronate
28.	Citrate cycle (TCA cycle)	1	Citrate
29.	Thiamine metabolism	1	Glycine
30.	Biosynthesis of unsaturated fatty acids	1	(4Z,7Z,10Z,13Z,16Z,19Z)-Docosahexaenoic acid
31.	Pyruvate metabolism	1	Acetate
32.	Lipoic acid metabolism	1	Glycine
33.	Glycolysis / Gluconeogenesis	1	Acetate
34.	Glutathione metabolism	1	Glycine
35.	Inositol phosphate metabolism	1	D-Glucuronate
36.	Tryptophan metabolism	1	L-Tryptophan
37.	Arginine and proline metabolism	1	L-Proline
38.	Fatty acid degradation	1	omega-Hydroxy fatty acid;
39.	Cysteine and methionine metabolism	1	L-Methionine
40.	Amino sugar and nucleotide sugar metabolism	1	D-Glucuronate
41.	Fatty acid biosynthesis	1	Octanoic acid

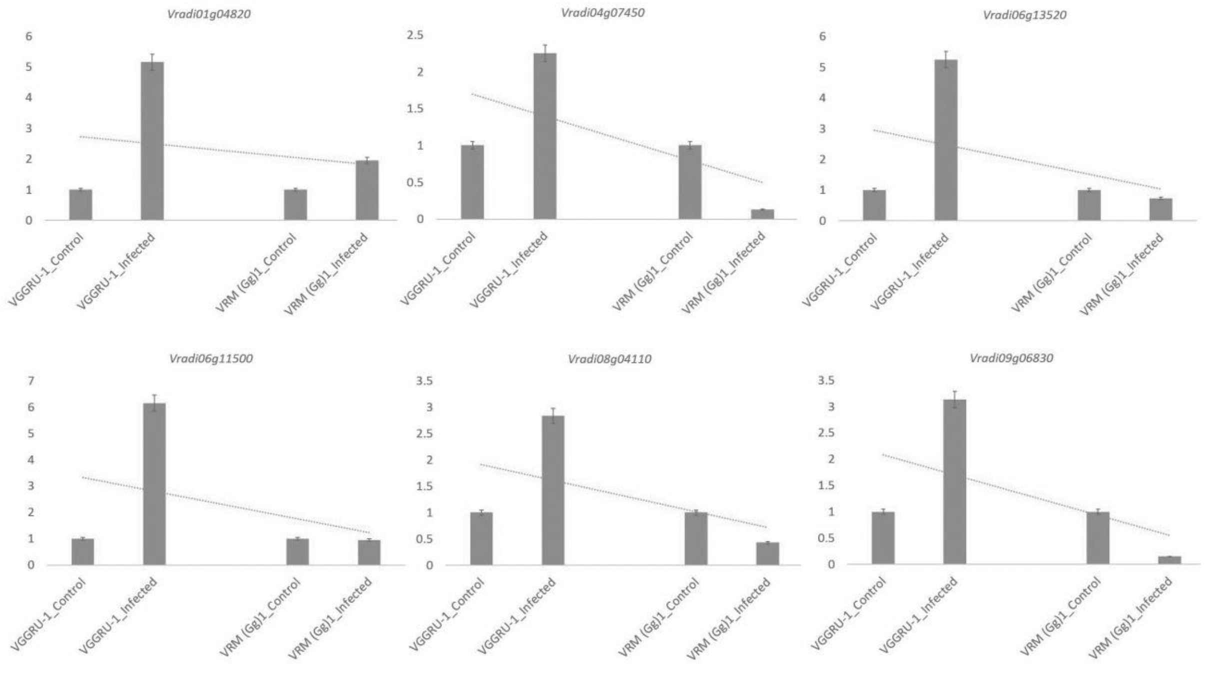

(Madhumitha *et al*., 2024)

Supplementary Figure S1. Graphs denote qRT-PCR-based validation of DEGs of mungbean leaves infected with mungbean yellow mosaic virus. The y-axis denotes relative fold change compared to control and infected. Data represent means ± SD of three replicates.
